# Comparative effects of amlodipine and benazepril on Left Atrial Pressure in Dogs with experimentally-induced Mitral Valve Regurgitation

**DOI:** 10.1186/1746-6148-8-166

**Published:** 2012-09-18

**Authors:** Shuji Suzuki, Ryuji Fukushima, Taisuke Ishikawa, Yuta Yamamoto, Lina Hamabe, Soomin Kim, Rieko Yoshiyuki, Noboru Machida, Ryou Tanaka

**Affiliations:** 1 Department of Veterinary Surgery, Faculty of Veterinary Medicine, Tokyo University of Agriculture and Technology, Tokyo, Japan

## Abstract

**Background:**

One of the purposes of treatment for dogs with mitral regurgitation (MR) is lowering left atrial pressure (LAP). There has been few study of the amlodipine in dogs with MR and amlodipine’s effect on LAP has not been fully evaluated in a quantitative manner because of difficulties in directly measuring LAP. The objective of our study was to compare the short-term effects of amlodipine (0.2 mg/kg PO q12h) vs benazepril (0.5 mg/kg PO q12h), on LAP and echocardiographic parameters in five beagle dogs with experimentally-induced MR. LAP of eight dogs that has own control were measured using radiotelemetry system at baseline and again on days 1, 2, 3, 4, 5, 6, 7 of the drug administration.

**Results:**

Mean LAP decreased significantly after amlodipine (11.20 ± 4.19 mmHg vs 14.61 ± 3.81 mmHg at baseline, p < .01) but not after benazepril treatment (13.19 ± 3.47 mmHg, p > .05). LAP was lower after 7 days of amlodipine treatment than after 7 days of benazepril treatment. Significant reduction was seen for the first time 4 days after the administration amlodipine. The rate of the maximal area of the regurgitant jet signals to the left atrium area (ARJ/LAA) of the amlodipine treatment was significantly lower (p < .05) after 7 days compared to baseline. Other echocardiographic parameters did not change significantly.

**Conclusions:**

LAP was significantly decreased after amlodipine treatment in dogs with surgically-induced MR but not after benazepril treatment. Although this study did not focus on adverse effects, amlodipine may be an effective drug for helping the patients with acute onset of severe MR, such as rupture of chordae tendinae or end stage patients were the LAP is likely to be elevated. Additional studies in clinical patients with degenerative mitral valve disease and acute chordal rupture are warranted because the blood-pressure lowering effects of amlodipine can decrease renal perfusion and this can further activate the RAAS.

## Background

Mitral valve disease (MVD) is the most common cardiac disease in dogs. Moreover, as many as 75% of all dogs with signs of congestive heart failure suffer from mitral regurgitation (MR) caused by myxomatous degeneration of the valve leaflets or chordae tendineae [1,2]. Left atrial compliance maintains a relatively normal left atrial pressure (LAP) in dogs with MR [3]. However, if left atrial compliance decrease, LAP will increase. Increased LAP causes pulmonary edema, which can lead to cough, dyspnea, and even death [4]. Reduction of LAP is a desirable goal for drugs used to treat congestive heart failure in MVD. In general, calcium channel blockers are used for treatment of hypertrophic cardiomyopathy [5-7], atrial fibrillation [8-10], and systemic hypertension [11,12]. Amlodipine is a long acting 1,4-dihydropyridine calcium channel blocker, often used for treatment of systemic hypertension. Systemic hypertension is defined as an increase of systolic and or diastolic blood pressure and common in dogs with renal disease and in aged and hyperthyroid cats [5]. In dogs with MR, amlodipine increases forward flow with afterload reduction because of the reduction in systemic vascular resistance [5,13]. Therefore, in theory, amlodipine are thought to decrease the volume of regurgitation across an insufficient valve and LAP. However, there has been few study of the amlodipine in dogs with MR and amlodipine’s effect on LAP has not been fully evaluated in a quantitative manner because of difficulties in directly measuring LAP. We have previously used a radiotelemetry system to report the effects of angiotensin-converting enzyme (ACE) inhibitors and furosemide in dogs with experimentally-induced MR [14-16], and we believe this system would be useful for evaluation of hemodynamic changes after administration of amlodipine.

In the present study, we used a radio telemetry system to monitor LAP in dogs with experimentally induced MR and evaluated the effects of amlodipine. Also we evaluated echocardiography, blood pressure (BP), and the effects of amlodipine on MR for analysis of pathophysiological changes.

## Methods

### Animals

Before starting the study, the health of five 2-year-old Beagle dogs (2 males and 3 females) weighing 12.4 ± 2.4 kg (range: 10.2 to 15.2 kg) was evaluated by general clinical examination, blood and serum bio chemical examinations, electrocardiography, thoracic radiography, and echocardiography. All dogs were acclimatized to the experimental environment and human handling. During all phases of the study, the dogs were managed and cared for in accordance with the standards established by the Tokyo University of Agriculture and Technology (TUAT) and described in its “Guide for the Care and Use of Laboratory Animals.” This study was approved by the Experimental Animal Committee of TUAT (acceptance no. 21–19).

### MR and transmitter implantation

Dogs were premedicated with meloxicam (Boehringer Ingelheim Vetmedica Japan, Hyogo, Japan) (0.2 mg/kg SC), atropine sulfate (Tanabe Seiyaku Co., Ltd., Osaka, Japan) (0.04 mg/kg SC), butorphanol tartrate (Meiji Seika kaisha Ltd., Tokyo, Japan) (0.2 mg/kg IV), and midazolam hydrochloride (Astellas Pharma Inc., Tokyo,Japan) (0.2 mg/kg IV). Induction was achieved with propofol (Schering-Plough, Tokyo, Japan) (4 mg/kg IV), after which the dog was intubated. General anesthesia was maintained with inhalation of isoflurane (Dainippon Sumitomo Pharma Co., Ltd., Osaka, Japan) mixed with oxygen. A left lateral thoracotomy was performed at the fifth intercostal space and the pericardium was opened by standard procedures. The left atrium was purse-string sutured with 3–0 nylon and a small incision was made at the center of the purse-string suture. The suture then was loosened and 5-in. curved Halsted mosquito forceps were inserted through the small incision to grasp and rupture the mitral valvular chordae tendineae. The position of the chordae tendineae and the degree of induced MR were monitored by transesophageal echocardiography and these procedures were repeated until visible MR was identified without any manual manipulation. The telemetry transmitter catheter (Data Sciences International, St. Paul, MN, USA) then was inserted 1 cm into the small incision and the catheter was fixed to the left atrium with a suture. The telemetry transmitter body was implanted under the triceps brachii muscle and the catheter was fixed to abdominal trunk muscles with 3–0 nylon suture. The chest then was closed in layers and air was evacuated by standard procedures. Postoperatively, cefamedin (Astellas Pharma Inc.) was administered (50 mg/kg/day IV or PO) for 7 days and postoperative pain was treated with meloxicam (0.1 mg/kg SC) for 3 days. Thoracic radiography and echocardiography were performed to evaluate pulmonary venous congestion and cardiac dilatation. After the radiotelemetry transmitter implantation, the dogs were rested for at least 5 weeks, until no major variations were identified in echocardiographic evaluation and LAP.

### Drug administration and LAP measurement

**Figure 1:**
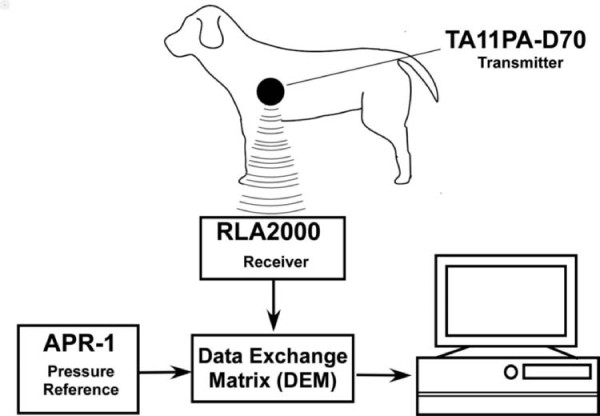
**The scheme of left atrial pressure measurement by a radio telemetry system in this experiment.** The digital signal of the data of the left atrial pressure from the transmitter was sensed by the receiver and sent to a data exchange matrix. An ambient pressure reference monitor was connected to the data exchange matrix to monitor correct atmospheric pressure and exclude all rocal environmental pressure fluctuations. Finally, the digital signal data was converted to an analog signal by the data exchange matrix and sent to a personal computer.

Amlodipine (Dainippon Sumitomo Pharma) of 0.2 mg/kg was administered PO q12h or benazepril (Novartis Pharma K.K., Tokyo, Japan) of 0.5 mg/kg was administered PO q12h to 5 dogs for 7 days. After a 7-day washout period, the other drug was administered for 7 days, using a crossover study method. All radiotelemetry systems (Data Sciences International) and recording procedures were the same as those described in a previous report [14]. The maximum, mean, and minimum LAP were obtained as the averages of 10-second segments from continuous waveform recordings (Figure 1) [17,18]. LAP was measured for 30 minutes from 21.00 to 21.30 at baseline and again on days 1, 2, 3, 4, 5, 6, 7 of the drug administration. The sampling frequency was every 10 seconds.

### Echocardiography

Before and after administration of each drug, echocardiographic measurements were performed along with blood pressure and LAP measurements. A single investigator performed transthoracic conventional echocardiography as well as 2-dimensional spectral Doppler and tissue Doppler echocardiography. Each dog was positioned in left and right recumbency, with echocardiographic examinations performed by means of a digital ultrasonographic system (Aloka Co., Ltd., Tokyo, Japan) with a 5.0 MHz sector transducer. Sweep speed during the Doppler and M-mode recordings was 150 to 200 mm/sec. Right parasternal views were used to measure heart dimensions. LA/Ao was assessed in a right parasternal short-axis view of the heart base for assessing LA enlargement [19]. We measured the internal diameter of the aorta along the commissure between the noncoronary and right coronary aortic valve cusp and internal diameter of the left atrium in a line extending from and parallel to the commissure between the noncoronary and left coronary aortic valve cusp to the distant margin of the left atrium on the 1^st^ frame after aortic valve closure. A short axis M-mode view at the chordal level was used to measure left ventricular end-diastolic diameter (LVEDD). Left apical views of the left ventricular inflow and outflow tracts were used to measure mitral inflow and aortic flow using a pulsed wave sample volume of 4 mm. Forward stroke volume (SV) and cardiac output (CO) were calculated by use of a left ventricular outflow view. SV was calculated as SV = velocity time integral (VTI) × cross-sectional area (CSA). CO was calculated as CO = SV × HR. The systolic (Sa) and early diastolic (Ea) myocardial velocities by pulsed tissue Doppler imaging (TDI) were measured at lateral mitral annulus in the left apical views. Peak transmitral early diastolic wave (E wave) velocity and atrial contraction wave (A wave) were measured from Doppler signals of the mitral inflow, and E/A, E/Ea were calculated. MR flow was recorded using the 2-chamber view with a high-intensity continuous wave spectral Doppler signal, and MR pressure gradient was calculated based on a modified Bernoulli’s equation, ΔP = 4(MR velocity)^2^. The measurements of the maximal area of the regurgitant jet signals (ARJ) was performed in the apical 4-chamber view, the left atrium area (LAA) also was measured in the same frame in the which the maximal ARJ was seen, and ARJ/LAA was calculated. Maximum color Doppler velocity was set at 66 cm/s. Color gain was decreased until background noise just disappeared [20]. All echocardiographic variables were averaged from 5 consecutive beats. All of the echocardiographic recordings were stored on the internal hard drive of the echocardiograph and transmitted to the DICOM server online (ImageONE Co., Ltd., Tokyo, Japan).

### Blood pressure measurements

All indirect arterial BP recordings were obtained by the oscillometric method (Fukuda ME, Tokyo, Japan). Cuff size width was set to approximately 40% of tail circumference for each dog [21]. BP measurements were performed simultaneously with echocardiography for MR velocity measurements, and 5 consecutive measurements were averaged for each dog for use in the calculations. Systemic vascular resistance (SVR) was calculated as SVR = 79.9 × (Mean BP-Central venous pressure)/CO [22], and Cemtral venous pressure was defined as 5 mmHg due to a lack of right-sided heart failure signs, jugular distension, or positive hepatojugular reflex.

### Statistical analysis

All data are represented as mean plus or minus standard deviation (SD). All data were normally distributed. A 1-way repeated measures analysis of variance (ANOVA) in conjunction with a Bonferroni’s multiple comparison test was used for comparing LAP and echocardiographic variables before and 7 days after administration of each dose. A 2-way repeated measures ANOVA in conjunction with a Bonferroni’s multiple comparison test was used to compare LAP of each day after the administration of amlodipine. Statistical significance was defined as p < .05. GraphPad Prism version 5.0a (GraphPad, San Diego, CA, USA) and EXCEL 2008 (Microsoft, Redmond, WA, USA) were used to perform these statistical analyses.

## Results

### LAP and blood pressure

All dogs were stage B2 in guidelines for the diagnosis and treatment of canine chronic valvular heart disease of the American College of Veterinary Internal Medicine (ACVIM) classification [23]. No obvious adverse effects were observed during periods of the drug administration.

**Figure 2:**
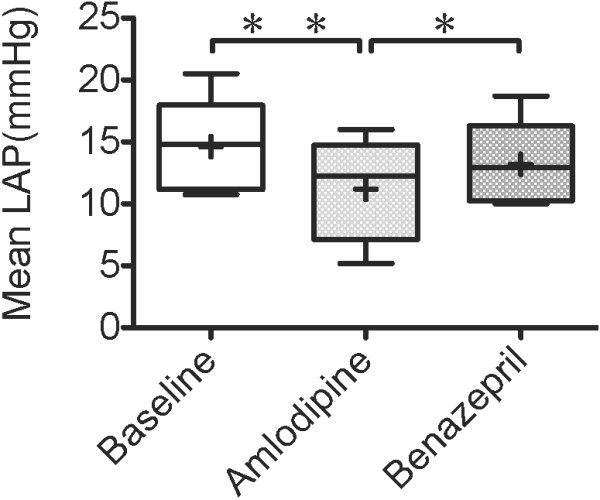
**Left atrial pressure 7 days after administration of amlodipine.** Left atrial pressure (LAP) 7 days after administration of amlodipine 0.2 mg/kg or benazepril 0.5 mg/kg q12h PO in 5 dogs with MR. Baseline values were those obtained before drug administration. The box and whiskers plot demonstrates the mean (+), the median (line), 5 and 95% confidence limits (box) and range (bars). ^*^P < .05, ^**^P < .01.

Mean LAP decreased significantly after the administration of amlodipine (14.61 ± 3.81 mmHg to 11.20 ± 4.19 mmHg, p < .01), as shown in Figure 2. Similarly, Mean LAP decreased after the administration of benazepril, however, there was no significant difference (14.53 ± 3.92 mmHg to 13.19 ± 3.47 mmHg, p > .05). Also, LAP was lower after 7 days of amlodipine treatment than after 7 days of benazepril treatment (p < .05).

**Figure 3:**
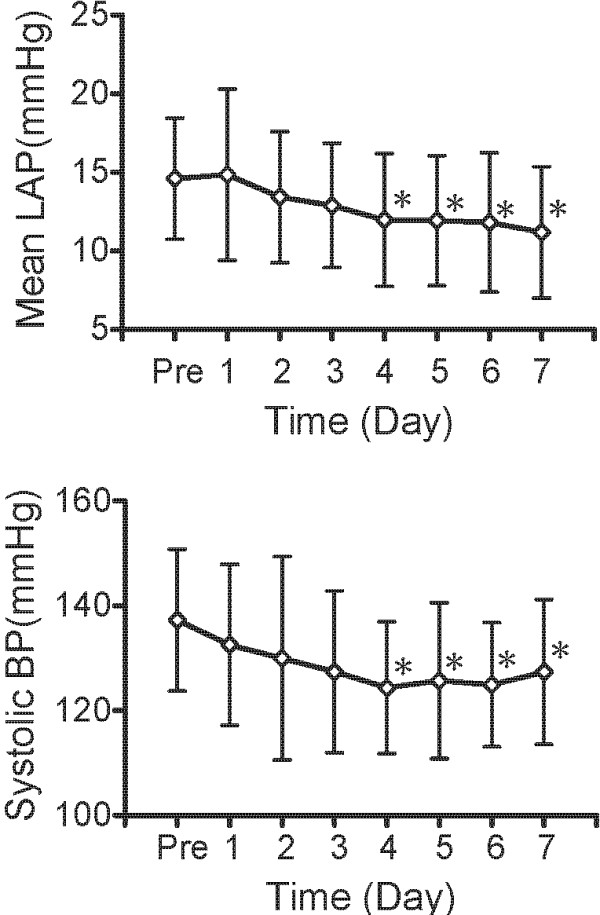
**Temporal variation after administration of amlodipine.** Temporal variation of Mean LAP and Systolic blood pressure after administration of amlodipine in 5 dogs with MR. ^*^Significant differences (P < .05) compared with each baseline.

**Figure 4:**
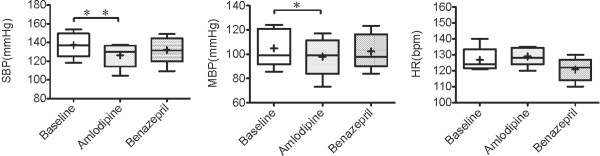
**Systolic blood pressure Mean blood pressure and Heart rate 7 days after administration of amlodipine.** Systolic blood pressure (SBP), Mean blood pressure (MBP) and Heart rate (HR) 7 days after administration of amlodipine 0.2 mg/kg or benazepril 0.5 mg/kg q12h PO in 5 dogs with MR. Baseline values were those obtained before drug administration. The box and whiskers plot demonstrates the mean (+), the median (line), 5 and 95% confidence limits (box) and range (bars). ^*^P < .05, ^**^P < .01.

Statistically significant reduction of LAP was first observed 4 days after the administration of amlodipine, as shown in Figure 3. Similarly, significant reduction of SBP was first observed 4 days after the administration of amlodipine. SBP and MBP of both the amlodipine and benazepril treatment decreased significantly compared to baseline, as shown in Figure 4. Also, SBP was lower after 7 days of amlodipine treatment than after 7 days of benazepril treatment, however, there was no significantly difference. HR of both the amlodipine and the benazepril treatment did not change significantly as shown in Figure 4.

### Echocardiography and hemodynamic parameters

**Figure 5:**
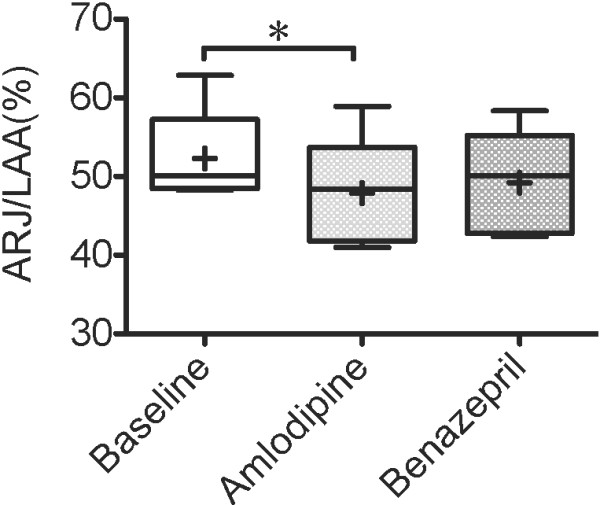
**ARJ/LAA 7 days after administration of amlodipine.** The ratio of the maximum area of the regurgitant jet signals to the left atrium area (ARJ/LAA) 7 days after administration of amlodipine 0.2 mg/kg or benazepril 0.5 mg/kg q12h PO in 5 dogs with MR. Baseline values were those obtained before drug administration. The box and whiskers plot demonstrates the mean (+), the median (line), 5 and 95% confidence limits (box) and range (bars). ^*^P < .05.

**Figure 6:**
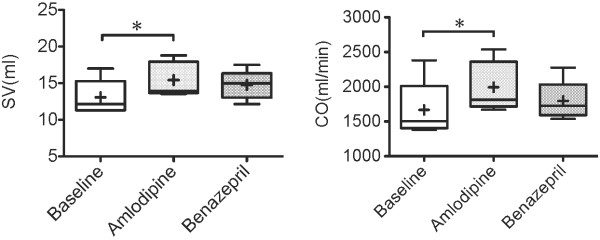
**Stroke volume and Cardiac output 7 days after administration of amlodipine.** Stroke volume (SV) and Cardiac output (CO) 7 days after administration of amlodipine 0.2 mg/kg or benazepril 0.5 mg/kg q12h PO in 5 dogs with MR. The box and whiskers plot demonstrates the mean (+), the median (line), 5 and 95% confidence limits (box) and range (bars). ^*^P < .05.

**Figure 7:**
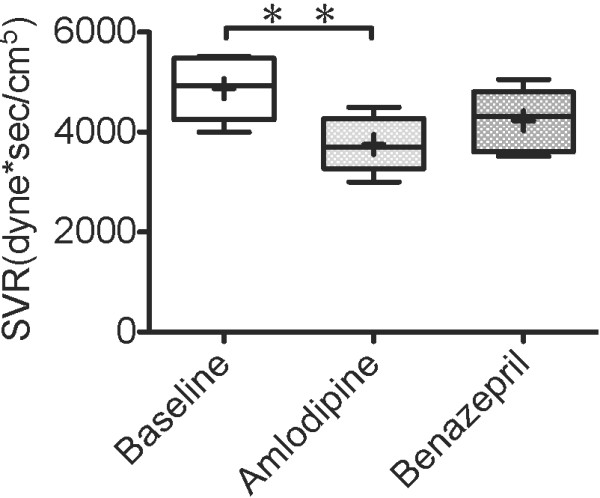
**Systemic vascular resistance 7 days after administration of amlodipine.** Systemic vascular resistance (SVR) 7 days after administration of amlodipine 0.2 mg/kg or benazepril 0.5 mg/kg q12h PO in 5 dogs with MR. The box and whiskers plot demonstrates the mean (+), the median (line), 5 and 95% confidence limits (box) and range (bars). ^**^P < .01.

ARJ/LAA was significantly lower after 7 days of amlodipine treatment than baseline (p < .05), as shown in Figure 5. As shown in Table 1, other echocardiographic parameters did not change significantly. SV and CO of the amlodipine treatment increased significantly compared to baseline, as shown in Figure 6. SVR of amlodipine treatment decreased significantly compared to baseline, as shown in Figure 7.

**Table 1:** Comparison of echocardiographic parameters after the drug administration

**Variable**	**Baseline**	**Amlodipine**	**Benazepril**
LVEDD (cm)	3.77 ± 0.33	3.65 ± 0.25	3.58 ± 0.36
LVESD (cm)	2.30 ± 0.15	2.09 ± 0.20	2.16 ± 0.27
%FS	38.7 ± 4.68	41.7 ± 6.00	39.8 ± 7.02
LA/Ao	1.54 ± 0.29	1.51 ± 0.17	1.60 ± 0.14
E wave (m/sec)	0.85 ± 0.13	0.81 ± 0.08	0.82 ± 0.10
E/A	1.79 ± 0.56	1.70 ± 0.21	1.76 ± 0.70
E/Ea	7.33 ± 1.58	7.16 ± 1.68	7.45 ± 1.72
Sa (cm/sec)	10.04 ± 1.07	12.72 ± 3.26	11.29 ± 2.62
MRPG (mmHg)	125.0 ± 12.5	130.6 ± 13.0	125.5 ± 10.8

See materials and methods section for the technique of measuring echocardiographic parameters. LVEDD, left ventricular internal diameter in the diastolic period; LVESD, left ventricular internal diameter in the systolic period;%FS, fractional shortening; LA/Ao, the ratio of left atrial diameter to aortic root diameter; E wave, transmitral early diastolic wave velocity; E/A, the ratio of transmitral early diastolic wave velocity to atrial contraction wave velocity; E/Ea, the ratio of transmitral early diastolic wave velocity to lateral mitral annulus velocity in the early diastolic period; Sa, lateral mitral annulus velocity in the systolic period; MRPG, mitral regurgitation pressure gradient.

## Discussion

This study had a number of important findings. Firstly, LAP was significantly decreased with amlodipine in dogs with MR. Secondly, LAP appeared to decrease according to the decreasing in SBP and MBP. Thirdly, stroke volume and cardiac output increased and systemic vascular resistance decreased after administration of amlodipine. Finally, the severity of mitral regurgitation appeared to decrease according to the reduction in ARJ/LAA.

In a previous report, amlodipine decreased left ventricular end-diastolic pressure (measured as a surrogate for LAP) in dogs with pacing-induced heart failure [13]. However, there are few studies that the effect of amlodipine on left atrial pressure in dogs with MR. In the present study, LAP was significantly decreased and cardiac output was significantly increased by amlodipine in dogs with MR (Figure 2 and 6). Amlodipine is arterial vasodilators and used to reduce systemic vascular resistance in patients with cardiac heart failure [5]. The reduction in systemic vascular resistance results in an increase in cardiac output. In dogs with MR, amlodipine increases cardiac out put, therefore, decreases the volume of regurgitation relatively across a mitral valve. So, our results suggest amlodipine has beneficial effect on LAP in dogs with MR. Also, in the present study, adverse effects of amlodipine were not observed but we feel this warrants further examination because there is a report that long-term administration of amlodipine to dogs with mitral regurgitation may cause gingival hyperplasia in a small percentage of patients [24].

Both of the amlodipine and benazepril are classified of vasodilators based on mechanism of action. Amlodipine was more effective than ACE inhibitor in lowering blood pressure [25]. In our previous report, an ACE inhibitor did not significantly decrease LAP despite a reduction in afterload [14]. Conversely, in the present study, amlodipine significantly decreased LAP, as well as SVR (Figure 7). This result suggests that strong reduction in afterload is associated with the decrease in LAP. Patients with heart failure need to maintain an adequate kidney perfusion and lowering the blood pressure per se can just induce renal failure (cardio-renal syndrome). So, while it is likely that amlodipine is an effective drug for helping the patients with acute onset of severe MR (i.e. rupture of chordae tendinae) or end stage patients were the LAP is likely to be elevated, the same cannot be true in patients stable chronic heart failure. It is thought that the positive long term benefits for patients treated with ACE inhibitor are related to their ability to block the Renin-Angiotensin-Aldosterone system (RAAS) more than to their vasodilation properties. On the other hand, indication of Amlodipine may be limited because the blood-pressure lowering effects of amlodipine can decrease renal perfusion and this can further activate the RAAS.

Amlodipine significantly decreased MBP and increased HR, although not statistically significant in previous reports [25]. Similarly, in the present study, amlodipine decreased MBP but did not change HR. The published dosage data in dogs is from 0.05 mg/kg/day [5] to 1 mg/kg/day [26]. The high end of this range (approximately 1 mg/kg/day) was chosen in the previous report [25]. However, in the present study, the dosage was 0.2 mg/kg q12h and HR might not have changed after administration of amlodipine. Further examination would be needed for the detailed information for the amlodipine dosage for dogs with MR.

There is a report on echocardiographic values of cats with systemic hypertension after administration of amlodipine [12]. However, no difference was found in any of the echocardiographic measurements between the untreated and treated cats in the previous report. In the present study, only ARJ/LAA of the amlodipine group was significantly lower compared to baseline (Figure 5). Other echocardiographic parameters did not change significantly. We previously have reported that E wave and E/Ea can be used for the evaluation of preload after administration of high doses of furosemide, and have monitored the reduction of LAP in the short-term [16]. However, present study suggests that it is difficult to evaluate the reduction of LAP after administration of amlodipine by using E wave and E/Ea. E, E/A and E/Ea are influenced not only by preload but relaxation, compliance, and heart rate [27]. Again it is the left atrial compliance that plays a major role in changing LAP. The echocardiographic indices might not have changed in this study because the left atrial compliance had been maintained despite the fluctuation of LAP. Conversely, ARJ/LAA, SBP and MBP may be useful to evaluation after administration of amlodipine.

Amlodipine is recommended at dosages of 0.05 mg/kg/day to 1 mg/kg/day for dogs [5,26]. However, the optimal dosage for dogs with MR is not known. This study was not designed to evaluate the optimal dosage of amlodipine for dogs with MR. Therefore, it may have some adverse effects by dosage. Also, toxic effects are not known with longterm administration of amlodipine in asymptomatic dogs because this study is shortterm study using dogs with experimentally-induced MR. In the present study, five 2-year-old Beagle dogs were used and a 5-week period was defined as a subchronic period for experimentally-induced MR. In clinical situations, dogs with MR and cardiac dysfunction and myocardial tissue damage might differ from the model dogs in this study. Therefore, our model may more closely resemble acute MR and differ from naturally-occurring chronic MR. SV and CO were calculated by echocardiography. Therefore, it may differ from the value strictly measured with the catheter.

In the present study, the neurohormonal response to treatment was not studied. Therefore, if a pure vasodilator is used to decrease blood pressure, there is a risk of a deleterious neurohormonal activation leading for example in increased heart rate.

## Conclusions

LAP was significantly decreased with amlodipine treatment in dogs with surgically-induced MR, while LAP was not significantly decreased with benazepril treatment. Although this study did not focus on adverse effects, amlodipine may be an effective drug for helping the patients with acute onset of severe MR such as rupture of chordae tendinae or end stage patients were the LAP is likely to be elevated. Additional studies in clinical patients with degenerative mitral valve disease and acute chordal rupture are warranted because the blood-pressure lowering effects of amlodipine can decrease renal perfusion and this can further activate the RAAS.

## Competing interests

The authors declare that they have no competing interests.

## Author’s contributions

SS, RF, TI, NM and RT participated in the conception and design of this study. SS, YY, LH, SK, RY and RT participated in the creation of experimentally-induced MR dogs. SS, YY and LH participated in the acquisition of data. SS, RF and RT participated in the analysis and interpretation of data. SS, NM and RT drafted the manuscript. All authors read and approved the final manuscript.
